# Methylation Marks of Blood Leukocytes of Native Hucul Mares Differentiated in Age

**DOI:** 10.1155/2019/2839614

**Published:** 2019-06-02

**Authors:** T. Ząbek, E. Semik-Gurgul, T. Szmatoła, A. Gurgul, A. Fornal, M. Bugno-Poniewierska

**Affiliations:** ^1^National Research Institute of Animal Production, Balice 32-083, Poland; ^2^University Centre of Veterinary Medicine, University of Agriculture in Kraków, Al. Mickiewicza 24/28, 30-059 Kraków, Poland; ^3^Institute of Veterinary Sciences, University of Agriculture in Krakow, 31-120, Poland

## Abstract

Horses are one of the longest-living species of farm animals. Advanced age is often associated with a decrease in body condition, dysfunction of immune system, and late-onset disorders. Due to this, the search for new solutions in the prevention and treatment of pathological conditions of the advanced age of horses is desirable. That is why the identification of aging-related changes in the horse genome is interesting in this respect. In the recent years, the research on aging includes studies of age-related epigenetic effects observed on the DNA methylation level. We applied reduced representation bisulfite sequencing (RRBS) to uncover a range of age DMR sites in genomes of blood leukocytes derived from juvenile and aged horses of native Hucul breed. Genes colocated with age-related differentially methylated regions (age DMRs) are the members of pathways involved in cellular signal transduction, immune response, neurogenesis, differentiation, development, and cancer progression. A positive correlation was found between methylation states and gene expression in particular loci from our data set. Some of described age DMR-linked genes were also reported elsewhere. Obtained results contribute to the knowledge about the molecular basis of aging of equine blood cells.

## 1. Introduction

Aging is associated with accumulation of undesired physiological changes that finally lead to a functional decline. Due to the fact that breeding horses are fairly long-living species of animals, this requires a search for any new solutions in the prevention and treatment of late-onset diseases. Horses at the age over 20 years constitute to nearly 15 percent of the whole population being actively used in riding and reproduction. However, advanced age in horses associates with a decrease in body condition and dysfunction of the immune system. The immune impairment of aged horses is expressed by a decrease in proliferative potential of T cells and diminished antibody production in response to vaccinations [1]. Other examples of age-related diseases in horses include arthritis, congestive heart failure, and cancer [2]. It was found that certain epigenetic marks coincide with biological processes relevant for aging. DNA methylation is the most stable and mitotically heritable epigenetic trait which relies on the addition of the methyl group to the nucleotides. This kind of epigenetic modification plays a part in the regulation of gene expression. In this context, it refers primarily to GC-rich regions which feature promoters and regulatory elements of genes [3]. In the classical theory, DNA methylation is responsible for the blockage of gene expression and strongly correlates with the state of chromatin packing [3]. It is believed that DNA methylation conforms a link between the environment and the ongoing alterations of genome activity. The evident examples are studies which showed correlation between age-related hypomethylation with increased activity of the IFNg gene during maturation of the adaptive immune system of juvenile horses [4]. According to this, some of inherited methylation patterns established at the initial stages of embryo development could be gradually changed in the course of the lifespan. Such age-related methylation changes could be simply a random event or could have implications on particular phenotypes. As the example, a range of differentially methylated genes were assigned to methylation QTLs of biological aging in humans [5, 6]. Moreover, the set of 353 differentially methylated CpG sites has been proposed to serve as the fast perfect predictor of chronological age in numerous human tissues and blood [7].

The goal of the conducted study was the search against methylome differences of equine blood investigated in the healthy Hucul mares of different ages. The study included blood samples of juvenile, middle-aged, and senior horses of this breed. These primitive horses are of local pony breed which have been developed in mountainous conditions and are characterized with great endurance and hardiness [8]. Due to this, Huculs might be a valuable subject of studies on immunology and longevity in horses. The application of bisulfite sequencing of the portion of the horse genome enriched in CpG sites allowed us to find age-related methylation marks of horse blood leukocytes. A part of identified age DMR sites were associated with genes common for aging. The transcriptional profile of some of them correlated with methylation status of DMR sites providing the link between differential methylation and gene expression patterns. Significant alterations of methylation profiles at particular genomic locations in the juvenile-to-aged period might be relevant for aging of equine blood cells.

## 2. Materials and Methods

### 2.1. Biological Source

The sample set consists of blood samples from 6 juvenile Hucul fillies [82 H (7 mos), 76 H (8 mos), 77 H (8 mos), 78 H (8 mos), 85 H (8 mos), and 75 H (9 mos)], 3 middle-aged [35 H (13 yrs), 43 H (15 yrs), and 37 H (16 yrs)], and 3 senior mares [36 H (17 yrs), 40 H (18 yrs), and 42 H (20 yrs)] of this breed collected at the same day (5 ml of blood collected through the jugular venipuncture in vials coated with EDTA). Isolated DNA was used for RRBS library production and bisulfite conversion providing the templates for RRBS validation step. 3 ml of blood samples from horses of the second group of juvenile [4 H (2 mos), 5 H (2 mos), 6 H (1 mo), 7 H (3 mos), 8 H (3 mos), and 9 H (3 mos)] and aged Hucul mares [1 H (18 yrs.), 2 H (17 yrs.), 3 H (18 yrs.), 10 H (17 yrs.), 11 H (26 yrs.), 12 H (18 yrs.)] was collected in Tempus blood system vials (Life Technologies, Poland) and stored at -20°C for RNA preparation.

### 2.2. Reduced Representation Bisulfite Sequencing Approach

DNA was prepared from 1 ml of whole blood (Wizard Genomic DNA purification kit, Promega, Poland) using the manufacturer's recommendations. We then digested 100 ng of prepared DNA with methylation-insensitive restriction enzyme MspI to generate short fragments—each containing at least one CpG site. After end-repair, A-tailing, and ligation to methylated adapters with attached indexes, the CpG-rich DNA fragments were size-selected, subjected to bisulfite conversion, and PCR-amplified. Finally, we produced two pools of 6 bisulfite libraries which were quantified (Qubit fluorometer, Thermo Fisher Scientific, Poland) and evaluated for their quality (TapeStation system, Agilent, Poland). We applied 50 cycles of single-read sequencing by synthesis on the HiScanSQ system (Illumina, USA). We made 30% spike-in of PhiX Control DNA (Illumina, USA) to increase the nucleotide diversity of RRBS libraries during the first cycles of NGS run.

### 2.3. Processing of Bisulfite Sequencing Data

The trimming of low-quality base calls, sequence adapter removal, and inclusion of filtering against PCR duplication were applied to generate high-quality sequence reads (Flexbar software). Enriched CpG fractions of genomic DNA treated with bisulfite agent were the source of methylation measurement in the CG context. The assessment of absolute methylation levels relied on the percentage of cytosines and thymines at CG positions across all bisulfite sequencing reads aligned to the reference sequence. Base-by-base methylation calling was performed using at least ten reads of coverage, and NGS read alignment was made to the horse reference sequence EquCab 2.0 using BSMAP software. methylKit [9] was used to assess the quality of NGS read alignment and to identify age-related differentially methylated regions (age DMRs). We have applied a 1000 bp sliding window approach based on logistic regression and the use of Fisher's test.

### 2.4. Downstream Analysis Using Web-Available Bioinformatic Tools

Each of the determined age DMR was checked for the CG content using UCSC Table Browser (https://genome.ucsc.edu/cgi-bin/hgTables) relying on the EquCab 2.0 version of the horse genome. Variant Effect Predictor and the BioMart database available on the Ensembl website were applied for annotation of age DMRs against the EquCab2.0 reference sequence. In order to retrieve the biological context of age-related differential methylation of horse blood, we used tools available in PANTHER v 11.0 to make a molecular classification of age DMR-linked genes using horse genome as the background.

### 2.5. Validation of RRBS Results

12 DNA samples derived from the blood of both aging groups were the subject of cloned BSPCR sequencing. Bisulfite-converted DNA samples (EpiTect Bisulfite Conversion kit, Qiagen, Hilden, Germany) were amplified with the use of Hot Start Taq DNA Polymerase (Qiagen, Hilden, Germany) and the two-step PCR protocol including primer design for converted DNA sequence (Methyl Primer Express v 1.0 software). Selected age DMR fragments were the subject of cloned Sanger sequencing performed for 12 DNA samples used for RRBS experiment. The cloning procedure (TOPO TA Cloning Kit for Sequencing, Life Technologies, Poland) and SANGER sequencing (BigDye Terminator Sequencing chemistry, Life Technologies, Poland) were done under the manufacturer's recommendations. Methylation assessment for cloned BSPCR sequencing reads was conducted using BISMA software [10]. Observed variability of percent of methylation (PM) averaged at all CpG sites in the given DNA fragment was explained in the form of line charts with the correlation coefficient (*R* ^2^) between PM values supported by the trend line (plotted and calculated in Excel).

### 2.6. Assessment of Expression of Selected Genes with Associated Age DMRs

In order to discover potential relationships between age-related methylation and expression changes, the amount of transcripts of selected age DMR-linked genes was assessed using RNAs of blood samples derived from horses of the second group. The total RNA was isolated using MagMAX™ for Stabilized Blood Tubes RNA Isolation Kit (Life Technologies, Poland). The quantity and quality of RNA were examined by NanoDrop (Thermo Scientific, Poland) and by 2% gel electrophoresis. Reverse transcriptions were performed on 1 *μ*g of total RNA using SuperScript® VILO™ cDNA Synthesis Kit (Life Technologies, Poland) according to the protocol. RT-PCR primers were designed in the splice sites using Primer-BLAST [11]. The amount of mRNA levels of selected genes was evaluated on Eco Real-Time PCR System (Illumina, USA). Reactions (40 cycles) were performed in triplicates in a total volume of 10 *μ*l using RT-PCR primers and Fast EvaGreen Master Mix under the manufacturer's instructions (Biotium Inc., USA). Relative quantification for each gene was evaluated based on the SDHA gene used as appropriate endogenous control for equine blood [12]. To define the efficiency of real-time PCR, the standard curve method was performed for each gene. Quantification of mRNA levels was performed by the comparative ΔΔCT method according to Pfaffl [13]. *F* statistics with Welsh correction was implemented to assess the significance of fold change differences between juvenile and aged horse groups. Boxplots were used to show the differences of the fold change parameter (R software).

## 3. Results

### 3.1. Raw Data Characteristics

RRBS sequence reads were deposited in the SRA database under bioproject No. PRJNA517684. Libraries prepared after DNA digestion with MspI and bisulfite conversion brought on average 9.4 mln of high-quality reads per sample (since 4.7 to 14.5 mln) which gave 96% of input reads (since 4.8 to 14.9 mln). The results of mapping of bisulfite sequencing reads against the EcuCab2 version of the horse reference sequence revealed a big similarity of global methylation between blood samples (the high value of correlation coefficient of methylation percent between samples; Figure S1).

### 3.2. Description of Age DMR Sites

**Figure 1:**
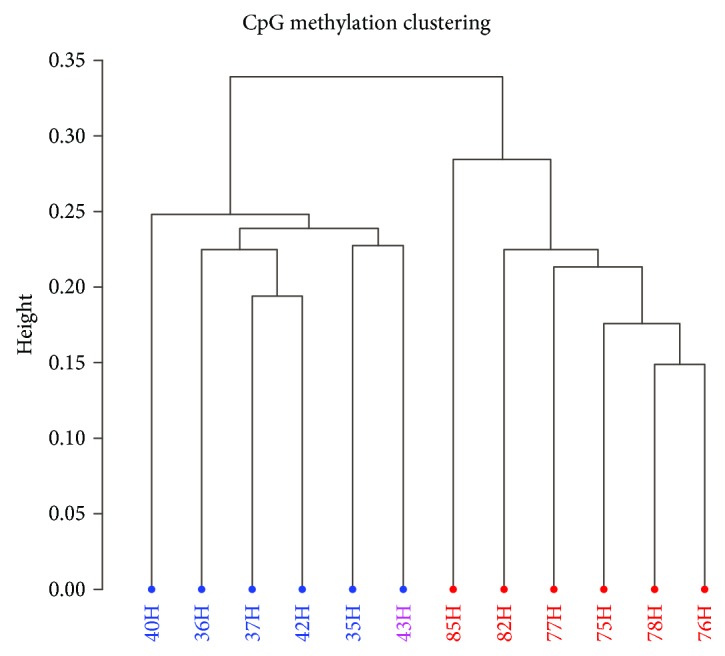
Hierarchical clustering (Ward method) of methylation profiles among 12 horse blood samples. The height denotes the distance between samples based on the degree of methylation differences detected in all differentially methylated CpG sites (DM CpGs) across the genome.

The initial stage of analysis showed the presence of near 10000 CpG sites whose methylation percent differed between both groups of horses. A dendrogram based on the correlation distance between samples revealed clear methylome clustering for blood samples of middle-aged and senior individuals (marked in blue) and for samples derived from juvenile horses (marked in red) (Figure 1).

Regions spanning differentially methylated CpG sites were distributed across 27 autosomes (except ECA 12, 21, 30, and 31). A single age DM CpG site was also found on ECAX. Highly significant differences between groups included 51 age-related differentially methylated regions of hypomethylation during the juvenile to aged period and 14 age DMR sites of the state of hypermethylation. Lowering the significance level to 0.05 allowed enlarging the list of 3 additional hypomethylated age DMRs (Table S1). Significant age DM CpG sites resided in 28 age DMR regions (sliding windows of 1 kbp in length) with CG content greater than 50%. The rest of them occurred in regions with the mean CG content of 42% (Table S1). 24 age DMR sites were found inside sequences of 22 coding loci (“gene bodies”), where 19 were located in introns (13 hypo- and 6 hypermethylated age DMRs) and 5 covered transcriptional units (2 hypo- and 3 hypermethylated age DMRs). The other identified age DMRs neighbored 68 coding loci being situated from 134 to 440035 bp upstream or downstream away from the nearest gene (Table S1).

### 3.3. Characteristics of Encoded Proteins by Age DMR Collocated Genes

A substantial number of age DMR-linked loci found in this report encode membrane (EPHA7, SLC44A3, TMEM189, MMD2, RNF19B, ZPLD1, ST8SIA6, STAB2, STX2, KCND3, SDK2, LOC100054852, ACSL1, CACNA1C, TRAPPC2, IGF1R, AQP8, ATG5, GPNMB, and MDGA2) and signaling proteins (DAB1, GPNMB, GNL1, and TIAM1) important for cellular communication. Others are age DMR genes encoding transcription factors (TFs) (Table S2). Among them are important for the regulation of tissue- and cell type-specific gene transcription during development and adulthood (FOXP1) [14], TFs having common regulatory influence on a broad range of genes (LDB2) [15], TF DNA-binding factors specific to GC-rich sequences (GCFC2), or TFs highly expressed in the brain—especially in regions responsible for memory function (CAMTA1) [16].

### 3.4. Functional Classification of Age DMR-Linked Genes

PANTHER classification revealed that genes with collocated age DMR sites are involved in pathways relevant for signal transduction including CACNA1C (hypomethylated) responsible for general cellular interactions (5HT2-type receptor-mediated signaling pathway P04374), TMEM189 (hypomethylated) involved in innate immunity signaling (Toll receptor signaling pathway P00054), GRIK3 (hypomethylated) important for synaptic responses in the brain (metabotropic glutamate receptor group III pathway P00039), and SLC44A3 (hypomethylated) responsible for neural signal transmission to a wide range of tissues (nicotinic acetylcholine receptor signaling pathway P00044) (Table S2). Other age DMR-associated genes are important for the functioning of the immune system like IL13 (hypomethylated) involved in molecular pathways relevant for modulation of immune responses and immune cell development (interleukin signaling pathway P00036), RASGRP1 (hypomethylated) responsible for immune disturbance in aged individuals (heterotrimeric G-protein signaling pathway-Gq alpha and Go alpha-mediated pathway P00027), and PTPN11 (hypomethylated) providing the immune support for cancer progression (interferon-gamma signaling pathway P00035) (Table S2). Another group of age DMR loci encodes proteins engaged in the functioning of the nervous system like STX2 (hypomethylated) being important for neurosecretion (nicotinic acetylcholine receptor signaling pathway P00044), NTN1 (hypermethylated) relevant for axon development (axon guidance mediated by Slit/Robo P00008), and FBXW11 (hypermethylated) showing neuroprotective effects in the form of slower progression of Parkinson's disease (Hedgehog signaling pathway P00025). Some other age DMR-linked genes found in this report belong to pathways important for growth and developmental processes like DCP1B (hypomethylated) responsible for cell growth and differentiation (TGF-beta signaling pathway P00052), IGF1R (hypermethylated) inducing cell growth and survival (insulin/IGF pathway-protein kinase B signaling cascade P00033), FGF18 (hypermethylation) involved in regeneration of tissues (FGF signaling pathway P00021), and TIAM1 (hypermethylated)—a broadly active signaling protein being responsible for cell invasion, metastasis, and carcinogenesis (ras pathway P04393) (Table S2).

### 3.5. Interrelations between Age-Related Methylation and Gene Expression Patterns

**Figure 2:**
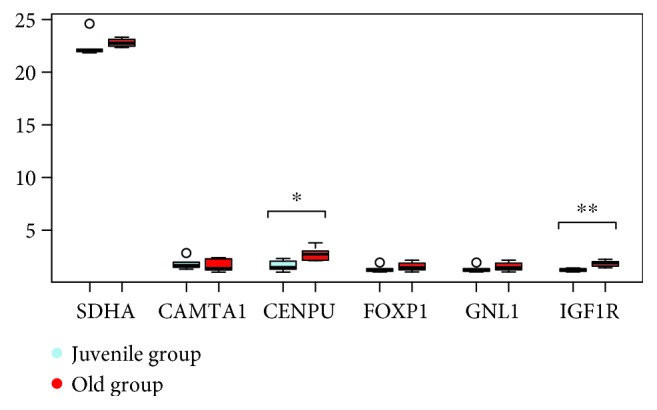
Box-and-whiskers plot of juvenile versus aged fold change in the expression of DMR-linked genes (◦*p* < 0.10, ∗*p* < 0.05, ∗∗*p* < 0.01, and ∗∗∗*p* < 0.001).

**Figure 3 fig3:**
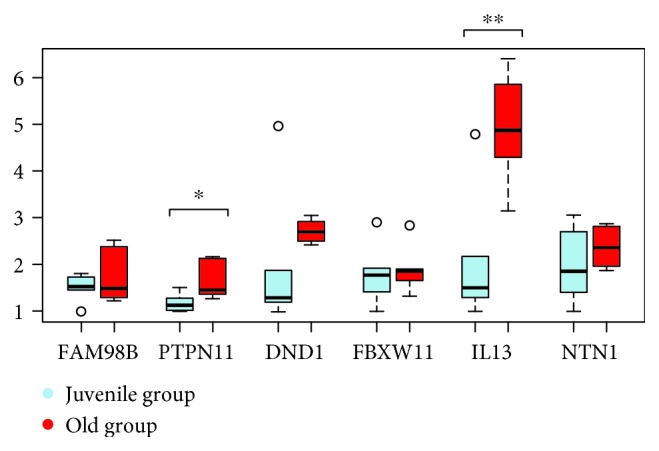
The follow-up of differential expression data of age DMR-linked genes (◦*p* < 0.10, ∗*p* < 0.05, ∗∗*p* < 0.01, and ∗∗∗*p* < 0.001).

**Figure 4 fig4:**
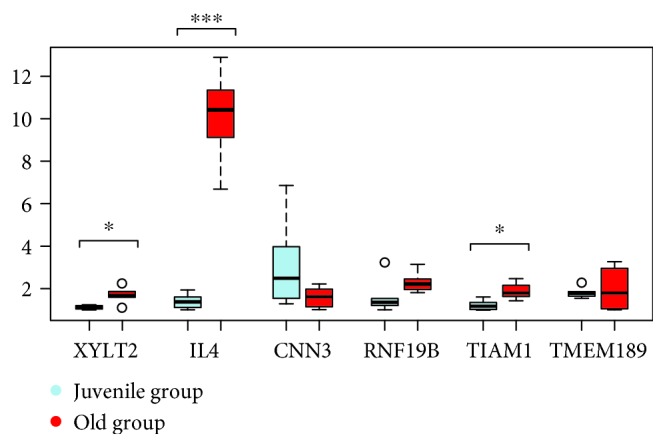
The follow-up of differential expression data of age DMR-linked genes (◦*p* < 0.10, ∗*p* < 0.05, ∗∗*p* < 0.01, and ∗∗∗*p* < 0.001).

**Figure 5:**
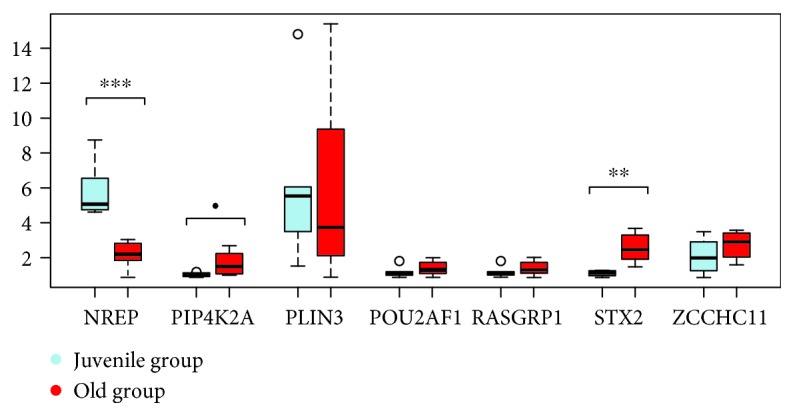
The follow-up of differential expression data of age DMR-linked genes (◦*p* < 0.10, ∗*p* < 0.05, ∗∗*p* < 0.01, and ∗∗∗*p* < 0.001).

24 coding loci (CAMTA1, CENPU, CNN3, DND1, FAM98B, FBXW11, FOXP1, GNL1, IGF1R, IL13, IL4, NREP, NTN1, PIP4K2A, PLIN3, POU2AF1, PTPN11, RASGRP1, RNF19B, STX2, TIAM1, TMEM189, XYLT2, and ZCCHC11) were the subject of real-time PCR using cDNA from blood of 6 juvenile and 6 aged Hucul mares (Figures 2 [Fig fig3] [Fig fig4]–5). Primer sequences used for RT-PCR are listed in Table S3. One hypermethylated (IGF1R) and four hypomethylated genes (IL4, IL13, NREP, and STX2) from our data set were differentially expressed with a high significance (Figures 2 [Fig fig3] [Fig fig4]–5). The expression differences for the other one hypermethylated (TIAM1) and three hypomethylated loci (CENPU, PTPN11, and XYLT2) were significant, and in case of a single gene (PIP4K2A), there was only a statistical trend. The hypomethylated state of upstream located age DMR sites in the juvenile to age period co-occurred with increased gene expression for seven loci (IL4, IL13, STX2, CENPU, PTPN11, XYLT2, and PIP4K2A) (Figures 2 [Fig fig3] [Fig fig4]–5). The opposite effect of methylation was the feature of the other 3 genes [age-related upregulation combined with gene body hypermethylation for the IGF1R and TIAM1 genes and the downregulation combined with hypomethylation of the 42500 bp upstream positioned DMR site in case of the NREP locus (Figures 2 [Fig fig3] [Fig fig4]–5)].

### 3.6. Sanger Sequencing Results of Age DMRs Typed by RRBS Experiment

**Figure 6:**
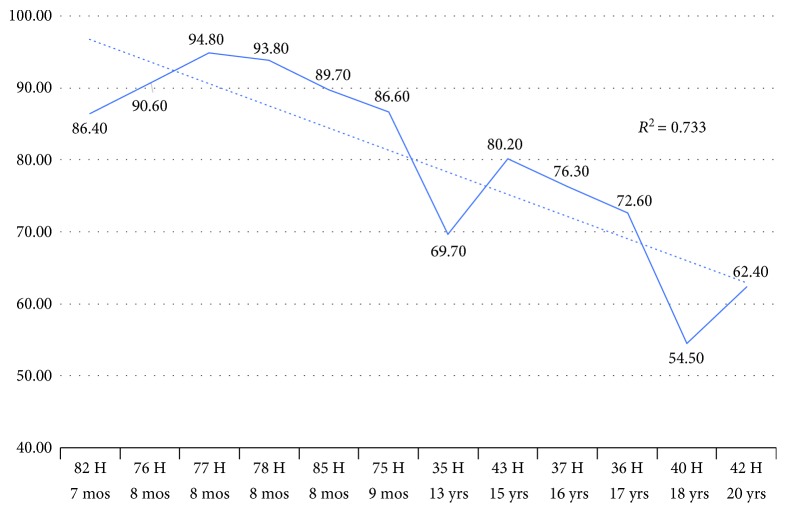
Line chart of averaged PM values across CpG sites of age DMR linked to the STX2 gene in blood samples of juvenile and aged group of horses.

**Figure 7:**
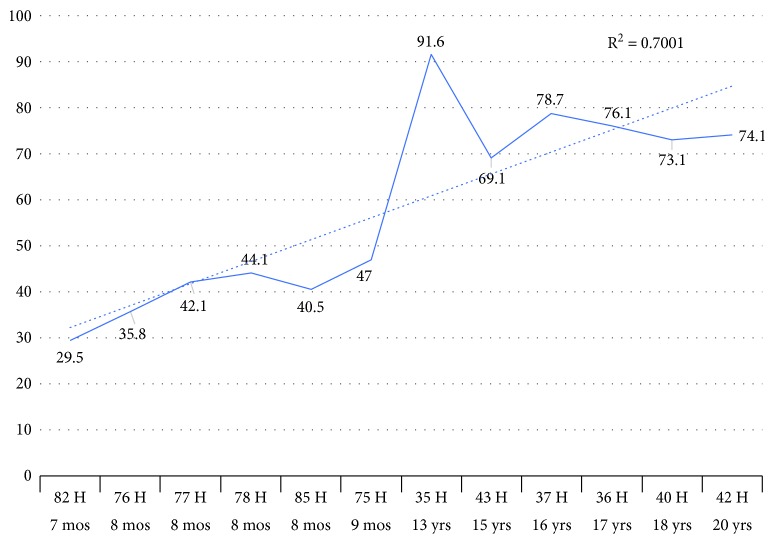
Line chart of averaged PM values across CpG sites of age DMR linked to the TIAM1 gene in blood samples of juvenile and aged group of horses.

The methylation state of two DNA fragments showing hypo- (age DMR proximal to STX2) and hypermethylation (intronic age DMR of TIAM1) in the juvenile to aged periods were verified through cloned bisulfite sequencing (Table S4). Sanger sequencing confirmed the observed tendency of methylation differences between both aging groups of Hucul horses in reference to the RRBS results. A positive trend (high *R*-square values) of observed decrease (*r* ^2^ = 0.7333) or increase in methylation percent (*r* ^2^ = 0.7001) linked to the age of blood samples was shown on the line charts of Figures 6 and 7, respectively. Certain deviations from the observed trends of PM values refer to the potential variability of blood cell proportions for particular horses included in the study (Figures 6 and 7).

## 4. Discussion

### 4.1. The Importance of Identified Age DMR Sites upon the Localization and Sequence Context

Ongoing hypo- and hypermethylation of DNA sequences during aging are two different aspects. A gradual reduction in methylation during aging is associated with the increase in defects of the genetic apparatus caused by the activity of transposable elements. Instead, hypermethylation seems to be more locus-specific in the processes of aging. For instance, methylation increase is often the trait of cancer development which is one of the functional hallmarks of aging. Mentioned age-related methylation changes affect in a majority of DNA sequences with a higher CG content which cover 2% of the whole genomic sequence, and such of those might be functionally important for processes associated with aging [17]. In the present report, the application of an MspI-cutting enzyme in the RRBS approach allowed us to characterize age-related differential methylation in the CG-enriched portion of the genome of horse blood leukocytes. A clear separate clustering of methylomes of juvenile horses from the older group is the proof of age-related effects visible on the DNA methylation level. A substantial part of identified age DMR sites in horse blood resided in CpG islands (CGIs). The number of hypomethylated age DMRs was substantially greater than that of hypermethylated ones in our data set coinciding with global loss of methylation during aging. However, many of the determined age DMR sites in the genome of equine blood leukocytes occupied the proximal regions of coding sequences or covered intronic or coding parts of genes. Moreover, considering the higher CG content of our collection of age DMRs, we assumed that many of them might in part serve as potential genomic sites of gene regulation.

### 4.2. Functional Background of Identified Age DMR Sites in the Genome of Horse Blood Leukocytes

Certain hypomethylated age DMR genes implicated in particular molecular pathways were relevant for neuronal signaling (GRIK3, SLC44A3, and STX2) and functioning of the immune system (TMEM189 and IL13) including immune disturbance in the old age (RASGRP1 and PTPN11). Gradual loss of methylation at upstream located age DMRs of immune-related genes might be the sign of increased gene expression relevant for impaired immune functions in the old age [18]. An also observed hypomethylation of immune-related genes might be linked to variable blood cell counts of myeloid and lymphoid lineage in individuals of different ages [19]. Moreover, age-related hypomethylation of genes involved in neural signal transduction observed in blood samples represents an interesting point for further research in horses. Horvath et al. [20] described a range of differentially methylated loci common for human blood and brain tissue being relevant for nervous system development, neuron differentiation, and neurogenesis. The authors concluded that blood could be an interesting target for studying aging-related alterations of methylome of nervous tissue. Two of hypermethylated age DMR loci from our data set were also important for neuronal functioning including axon development (NTN1) and neuroprotective proteolytic activity (FBXW11). This finding also strongly supports the idea about the blood as surrogate for studies on methylation-based neuronal effects during aging [20]. Other hypermethylated age DMR genes from our data set classified two pathways relevant for growth and development (DCP1B, IGF1R, and FGF18) and cancer progression (TIAM1)—biological processes which has been also mentioned in the literature as epigenetic hallmarks of aging [7]. Hypermethylation of equine blood leukocytes during aging might be then relevant for gene downregulation influencing impairment of cell differentiation, tissue regeneration, and neuronal decline. However, because many of hypermethylated age DMRs found in our study were located in gene bodies, their methylation state could also have a reciprocal effect on gene expression [21].

### 4.3. Examples of Interrelations between Age DMR Methylation and Relevant Gene Expression

The results of functional studies showed that DNA methylation of regulatory elements promotes blockage of gene expression. Epigenetic research focused on the aging of methylomes revealed that this relation is however more complicated [19]. It has been found that differential expression of a rather small number of loci truly correlated with differential methylation. Three of hypomethylated DMR sites in our study are located in the upstream positioned CpG islands (close to the transcriptional start site) of loci characterized by a significant increase in gene expression in aged horses (CENPU, IL4, and STX2). Mentioned age DMR genes would provide strong evidence for the link between methylation decrease and aging-related gene upregulation laying the foundation for functional studies. In turn, the importance of hypomethylation of nearly 40 kbp upstream the located DMR site to the NREP locus seems to be in contradiction to the above results. It was inversely associated with a highly significant downregulation of NREP in aged horses. The hypomethylated state of this age DMR is probably associated with differences of blood cell proportions between juvenile and adult horses and not with aging per se. The minority of identified DMR sites in the genome of equine blood leukocytes affected hypermethylation in the juvenile-to-aged period in our study. Two of them were found inside the boundaries of genes significantly upregulated in the blood of aged horses (IGF1R and TIAM1). The presence of such interrelations consists with growing evidence of a positive correlation between “gene body” methylation and gene expression where the methylation increase is crucial for gene upregulation [21]. As an example of potential significance of the observed methylation/expression relationships, it is the age-related upregulation of the CENPU gene in horse blood leukocytes. It was hypothesized that the CEN family of proteins might be implicated in structural genomic defects promoting cancer development [22]. The CENPU gene is one of factors required for centromere assembly during replication events being expressed on the constant level in normal cells [23]. However, overexpression of CENPU promotes proliferation of tumor cells through reduced activity of its transcription factor [24].

### 4.4. Descriptive Characteristics of Identified Age DMR Loci Based on Their Relevance for Aging Research

Many of age DMR-associated genes found in this study are similar to those known to be important genetic factors of aging and late-onset diseases. Among them, IGF1R triggers cell growth and survival [25]—processes tightly involved in aging and longevity. MRM1 encodes rRNA methyltransferase which when disturbed causes mitochondrial dysfunction leading to neural and immune impairment [26]. Genetic variants of POU2AF1 were associated with the occurrence of immune-senescence and autoimmune liver diseases [27]. Differential methylation of STAB2 was associated with pathophysiology of the cardiovascular system including pulmonary arterial hypertension [28]. And finally, a range of age DMR loci detected in horse blood (LDB2, SLC44A3, EPHA7, CAMTA1, MDGA2, FOXP1, TIAM1, NTN1, CNN3, EPHA7, GPR39, and ADAMTS5) are similar to those described in aging research of human blood leukocytes [20, 29]. It seems that epigenetic traits of aging could be conserved across mammals, and probably, the number of such age DMR loci will increase together with the growing interests in aging research in different species.

## 5. Conclusions

We have described age-related effects visible on the DNA methylation level of equine blood in primitive Hucul female horses. A substantial part of identified age DMR sites revealed hypomethylation in age horses. Certain genes linked to the loss of methylation were involved in pathways relevant for cellular signaling, including neural signal transduction and immunity. Age-related hypermethylation characterized genes implicated in development and differentiation being important for expansion of the neural network and cancer progression. A couple of genes found to be differentially expressed between both aging groups showed upregulation linked to methylation reduction in the aged group of horses. Inverted relations were detected for two upregulated genes in aged animals where hypermethylated age DMRs were located in introns. Some of the differentially methylated genes found in this study have been described in aging research in humans pointing on the presence of conserved epigenetic traits associated with chronological aging. The results of age-related differences of equine blood methylomes are novel for the epigenetic research of this mammalian species and contribute in general to the knowledge about the molecular factors of aging.

## Data Availability

RRBS sequence reads were deposited in the SRA database under bioproject No. PRJNA517684. The rest of the data are included in the manuscript and within the supplementary information files.

## Supplementary Materials

Supplementary Materials Figure S1: scatter plots (below the diagonal) and pairwise Pearson's correlation values (above the diagonal) of CpG methylation across 12 horse blood samples. Histograms on the diagonal denote the bimodal distribution of methylation patterns across all covered CpG sites. Table S1: characteristic of age DMR sites of horse blood leukocytes including localisation, differential methylation value, CG content, and annotation with the use of the EquCab 2.0 version of reference sequence. Table S2: molecular classification of age DMR-linked genes with the use of Panther v 11.0. Table S3: primer sequences applied for the RT-PCR approach. Table S4: calculation of methylation percent after clone bisulfite sequencing for age DMRs of the STX2 and TIAM1 genes.
