# Supplementary figures and images for: Methylation Marks of Blood Leukocytes of Native Hucul Mares Differentiated in Age
Source: Int J Genomics. 2019 Jun 2;2019:2839614. doi: 10.1155/2019/2839614 (PMC6589255; doi:10.1155/2019/2839614)

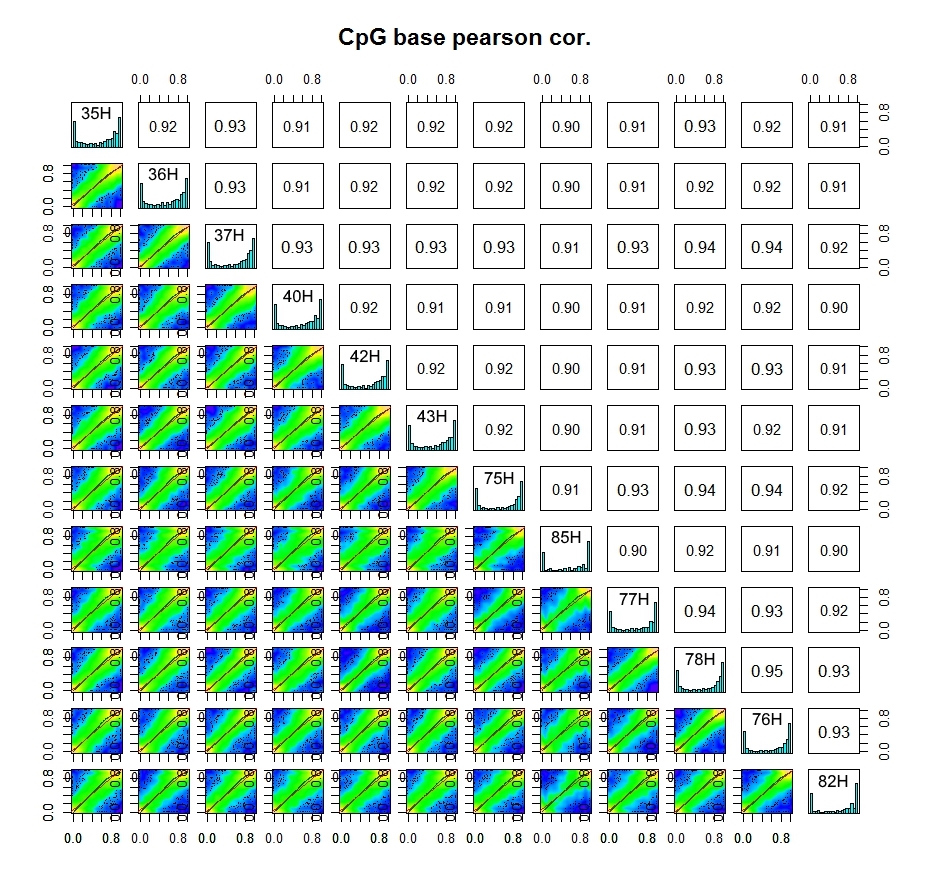

Supplement: Supplementary Materials — Figure S1: scatter plots (below the diagonal) and pairwise Pearson's correlation values (above the diagonal) of CpG methylation across 12 horse blood samples. Histograms on the diagonal denote the bimodal distribution of methylation patterns across all covered CpG sites. Table S1: characteristic of age DMR sites of horse blood leukocytes including localisation, differential methylation value, CG content, and annotation with the use of the EquCab 2.0 version of reference sequence. Table S2: molecular classification of age DMR-linked genes with the use of Panther v 11.0. Table S3: primer sequences applied for the RT-PCR approach. Table S4: calculation of methylation percent after clone bisulfite sequencing for age DMRs of the STX2 and TIAM1 genes. [file 2839614.f1.zip › Figure S1(1).jpeg]
